# Unsaturated fatty acids as immunometabolic modulators in high-risk pregnancies: mechanisms, clinical evidence, and translational perspectives

**DOI:** 10.3389/fphar.2026.1864831

**Published:** 2026-10-05

**Authors:** Edmarcio Olinto da Fonseca, Jocilda Carvalho de Faria, Regiane Penaforte Santos, Julia Vieira Carvalho, Caroline Maria Oliveira Volpe

**Affiliations:** Faculdade de Saúde Santa Casa BH, Programa de Pós-graduação Stricto sensu em Medicina/Biomedicina, Belo Horizonte, Brazil

**Keywords:** high-risk pregnancies, immunometabolism, inflammation, omega-3 fatty acids, placental dysfunction

## Abstract

High-risk pregnancies (HRPs) are characterized by the convergence of metabolic dysfunction, chronic low-grade inflammation, and placental maladaptation, leading to increased maternal and perinatal morbidity and mortality. Emerging evidence indicates that these conditions are driven by a tightly integrated immunometabolic axis, in which metabolic stress activates innate immune pathways, amplifying systemic and placental inflammation. Unsaturated fatty acids (UFAs), including monounsaturated and polyunsaturated fatty acids (PUFAs), have gained attention as key modulators of this axis. Omega-3 PUFAs, such as eicosapentaenoic acid (EPA) and docosahexaenoic acid (DHA), influence membrane composition, lipid mediator synthesis, and intracellular signaling pathways involved in inflammation and metabolism. At the cellular level, these fatty acids attenuate pro-inflammatory signaling through nuclear factor kappa B (NF-κB), modulate inflammasome activation, improve mitochondrial function, and promote the production of specialized pro-resolving mediators (SPMs), thereby facilitating the resolution of inflammation. Clinical evidence from observational studies, randomized controlled trials, and meta-analyses supports the role for omega-3 supplementation in reducing the risk of preterm birth, particularly among women with low baseline DHA status. However, findings for other outcomes, including preeclampsia and gestational diabetes, remain heterogeneous, likely reflecting variability in study design, baseline nutritional status, dosage, and timing of intervention. Although several studies support a beneficial role of omega-3 supplementation, the overall clinical evidence remains heterogeneous. While reductions in preterm birth are consistently observed, findings for other outcomes, such as preeclampsia and gestational diabetes, are inconsistent across randomized controlled trials. Differences in study design, baseline nutritional status, timing, dosage, and adherence likely contribute to these discrepancies. Importantly, several well-conducted trials have reported null effects, highlighting the need for cautious interpretation and more targeted approaches. This review synthesizes current evidence on the immunometabolic mechanisms underlying HRPs and highlights UFAs as biologically plausible and clinically relevant modulators. It focuses on the importance of precision nutrition approaches that incorporate baseline nutritional assessment and biomarker-guided stratification which may ultimately improve maternal and fetal outcomes in high-risk populations.

## 1 Introduction

High-risk pregnancies (HRPs) are characterized by the presence of maternal, fetal, placental, or contextual factors that increase the probability of adverse outcomes throughout pregnancy, delivery, and the puerperium. These conditions may originate from pre-existing disorders or arise during gestation, encompassing a broad spectrum of clinical scenarios, including gestational diabetes mellitus (GDM), hypertensive disorders such as preeclampsia, maternal obesity, and intrauterine growth restriction. Globally, HRPs are estimated to account for approximately 10%–20% of pregnancies and are consistently associated with elevated rates of maternal and perinatal morbidity and mortality (Holness, 2018; Zhang et al., 2025; Chavira-Suárez, 2025; Piron-Dumitrascu et al., 2025; Correa-de-Araujo and Yoon, 2021; Mirzakhani et al., 2020).

Successful pregnancy depends on the establishment of a highly regulated maternal–fetal interface, in which immune tolerance toward the semi-allogeneic fetus is balanced with controlled inflammatory responses (Albonici et al., 2020). This dynamic environment is supported by coordinated interactions between maternal immune cells and placental tissues, ensuring adequate placental development, vascular adaptation, and nutrient exchange. Disruption of this balance leads to immune dysregulation, placental dysfunction, and impaired fetal development (Rees et al., 2022; Khorami-Sarvestani et al., 2024).

Accumulating evidence indicates that HRPs are characterized by a state of chronic low-grade inflammation closely linked to metabolic disturbances, including insulin resistance, dyslipidemia, and oxidative stress. Rather than acting as independent processes, immune activation and metabolic dysfunction are increasingly recognized as components of an integrated immunometabolic axis that underlies the pathophysiology of these conditions (Figure 1) (Hajam et al., 2022; Rana et al., 2019; Anders et al., 2017; Catalano and Shankar, 2017).

**FIGURE 1:**
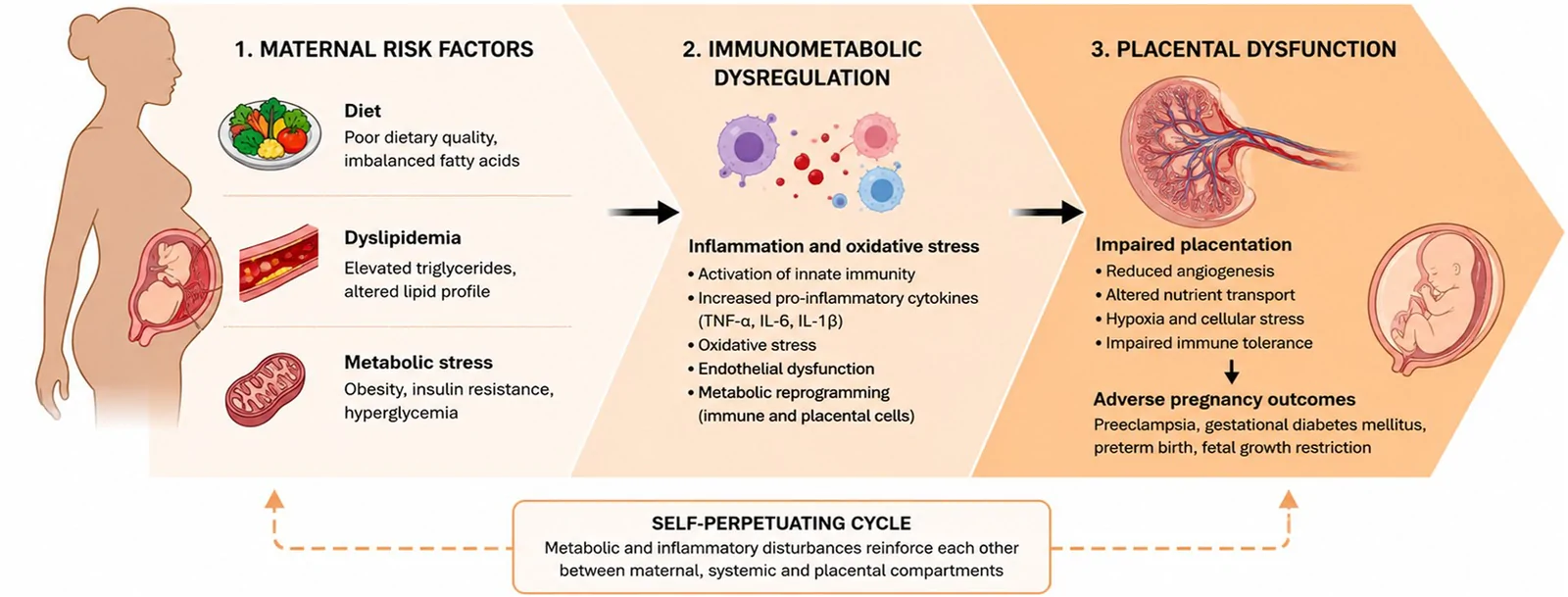
Immunometabolic axis in high-risk pregnancy. Maternal risk factors, including poor dietary quality and imbalanced fatty acid intake, dyslipidemia, obesity, insulin resistance, and hyperglycemia, initiate a cascade of interconnected metabolic and immune disturbances. These upstream factors promote immunometabolic dysregulation characterized by activation of innate immune pathways, increased production of pro-inflammatory cytokines (e.g., TNF-α, IL-6, and IL-1β), oxidative stress, endothelial dysfunction, and metabolic reprogramming of immune and placental cells. At the maternal–fetal interface, these alterations impair placentation by disrupting angiogenesis, trophoblast function, nutrient transport, oxygen homeostasis, and immune tolerance. The resulting placental dysfunction contributes to major adverse pregnancy outcomes, including preeclampsia, gestational diabetes mellitus, preterm birth, and fetal growth restriction. The figure also highlights the self-reinforcing nature of this immunometabolic network, in which metabolic stress, inflammatory signaling, and placental dysfunction interact in a bidirectional manner, perpetuating disturbances across maternal, systemic, and placental compartments.

In this context, nutritional factors have emerged as important modulators of immunometabolic homeostasis. Unsaturated fatty acids (UFAs), including monounsaturated fatty acids (MUFAs) and polyunsaturated fatty acids (PUFAs), play a central role in regulating membrane composition, lipid mediator synthesis, and intracellular signaling pathways involved in inflammation and metabolism. Omega-3 and omega-6 PUFAs serve as precursors of bioactive lipid mediators with distinct pro- and anti-inflammatory properties, thereby influencing both the initiation and resolution of inflammatory responses (Thompson et al., 2023; Sousa et al., 2025). Given their capacity to modulate key pathways involved in immune activation, oxidative stress, and metabolic regulation, UFAs have been proposed as biologically relevant modulators of the immunometabolic axis in pregnancy. This review aims to integrate current evidence on the mechanisms by which UFAs influence immunometabolic and placental function in HRPs, as well as to examine clinical evidence and discuss translational perspectives for targeted nutritional interventions.

## 2 Immunometabolic pathophysiology of HRP

HRPs arise from the interplay between pre-existing maternal conditions and inadequate adaptation to the metabolic demands of gestation. Although clinically heterogeneous, these conditions converge on a shared pathogenic framework characterized by chronic low-grade inflammation, oxidative stress, endothelial dysfunction, and impaired placental development (Burton and Jauniaux, 2018; Catalano and Shankar, 2017).

A central feature of this process is the activation of innate immune pathways in response to metabolic stress. Excess nutrient availability, lipotoxicity, and cellular dysfunction promote the release of endogenous danger signals that activate pattern recognition receptors (PRRs), including Toll-like receptors (TLRs). This triggers intracellular signaling cascades that converge on key inflammatory pathways, leading to sustained cytokine production and disruption of insulin signaling (Hotamisligil, 2017; Zindel and Kubes, 2020). Mitochondrial dysfunction further amplifies this response by increasing the generation of reactive oxygen species (ROS), which act as signaling molecules that reinforce inflammatory activation. In parallel, activation of inflammasome complexes, particularly the NLR family pyrin domain containing 3 (NLRP3) inflammasome, leads to the maturation and release of pro-inflammatory cytokines such as interleukin-1β (IL-1β), contributing to the persistence of sterile inflammation (Policastro et al., 2023; Rees et al., 2022).

Sterile inflammatory responses are also driven by alarmins, endogenous molecules released during cellular stress or damage. These include high-mobility group box 1 (HMGB1), uric acid, interleukin-1α (IL-1α), S100 calcium-binding protein B (S100B), heat shock proteins, and cell-free fetal DNA, which connect cellular stress to immune activation. These molecules act on placental and maternal immune cells to promote cytokine production, immune cell recruitment, and endothelial activation, contributing to a self-amplifying inflammatory loop (Brien et al., 2019; Zhang et al., 2024).

Within this context, the placenta functions as a central immunometabolic organ that integrates maternal metabolic signals with fetal developmental demands. Placental stress, driven by hypoxia, oxidative imbalance, and altered lipid metabolism, impairs trophoblast function and promotes the release of inflammatory mediators into the maternal circulation. This establishes a bidirectional crosstalk between systemic and placental compartments, further exacerbating inflammation and vascular dysfunction (Rana et al., 2019; Burton and Jauniaux, 2018).

Disturbances in maternal glucose homeostasis represent an additional and highly relevant component of immunometabolic dysregulation during pregnancy. In states of insulin resistance and hyperglycemia, particularly GDM, metabolic alterations directly influence immune cell function at the maternal–fetal interface. Excess glucose availability promotes a shift in placental macrophages toward a pro-inflammatory phenotype characterized by increased production of cytokines such as IL-1β, TNF-α, and IL-6, along with attenuation of regulatory responses. Recent evidence indicates that nutrient-derived signals, including both glucose and lipids, act as key modulators of innate immune pathways, highlighting the close integration between metabolic and immune regulation during pregnancy. These alterations contribute to placental inflammation, endothelial dysfunction, and adverse maternal and fetal outcomes (Giugliano et al., 2025; Barbosa et al., 2025; Mandò et al., 2024; Torres-Torres et al., 2024).

Taken together, these mechanisms define a self-sustaining immunometabolic network in which metabolic stress and immune activation are tightly interconnected, ultimately contributing to adverse outcomes such as preeclampsia, GDM, fetal growth restriction, and preterm birth (PTB).

## 3 Unsaturated fatty acids in pregnancy: mechanisms and functional implications

### 3.1 Structural and metabolic aspects of unsaturated fatty acids

UFAs are essential components of cellular membranes and play a central role in the regulation of metabolic and inflammatory processes. They are classified as MUFAs, which contain a single double bond, and PUFAs, which contain multiple double bonds and exhibit greater functional diversity (Dunbar et al., 2023; Calder, 2017; Benak et al., 2024).

Among PUFAs, omega-6 and omega-3 fatty acids are particularly relevant due to their role as precursors of bioactive lipid mediators. Because humans cannot synthesize the essential fatty acids linoleic acid (LA) and alpha-linolenic acid (ALA), these must be obtained through the diet and subsequently converted into longer-chain derivatives, including arachidonic acid (AA) from the omega-6 pathway and eicosapentaenoic acid (EPA) and docosahexaenoic acid (DHA) from the omega-3 pathway. The relative balance between omega-6 and omega-3 fatty acids is a key determinant of inflammatory homeostasis. A predominance of omega-6-derived substrates favors the synthesis of pro-inflammatory lipid mediators, whereas omega-3 fatty acids promote the generation of less inflammatory or pro-resolving mediators. This balance is particularly relevant in the context of modern dietary patterns, which are often characterized by disproportionately high omega-6 to omega-3 ratios (Djuricic and Calder, 2021; Duttaroy and Basak, 2022).

At the cellular level, AA is esterified into membrane phospholipids and released by phospholipase A2, subsequently serving as a substrate for cyclooxygenase (COX) and lipoxygenase (LOX) pathways. This results in the generation of predominantly pro-inflammatory eicosanoids, including prostaglandin E2 (PGE2) and leukotriene B4 (LTB4), which regulate leukocyte recruitment, vascular permeability, and amplification of inflammatory responses (Wei and Gronert, 2019; Cao et al., 2024). In contrast, omega-3 fatty acids, particularly EPA and DHA compete with AA for COX and LOX enzymes, generating lipid mediators that attenuate inflammation and actively promote its resolution through specialized pro-resolving mediators (SPMs), including resolvins, protectins, and maresins (Wei and Gronert, 2019; Calder, 2015; Serhan and Levy, 2018) (Figure 2).

**FIGURE 2:**
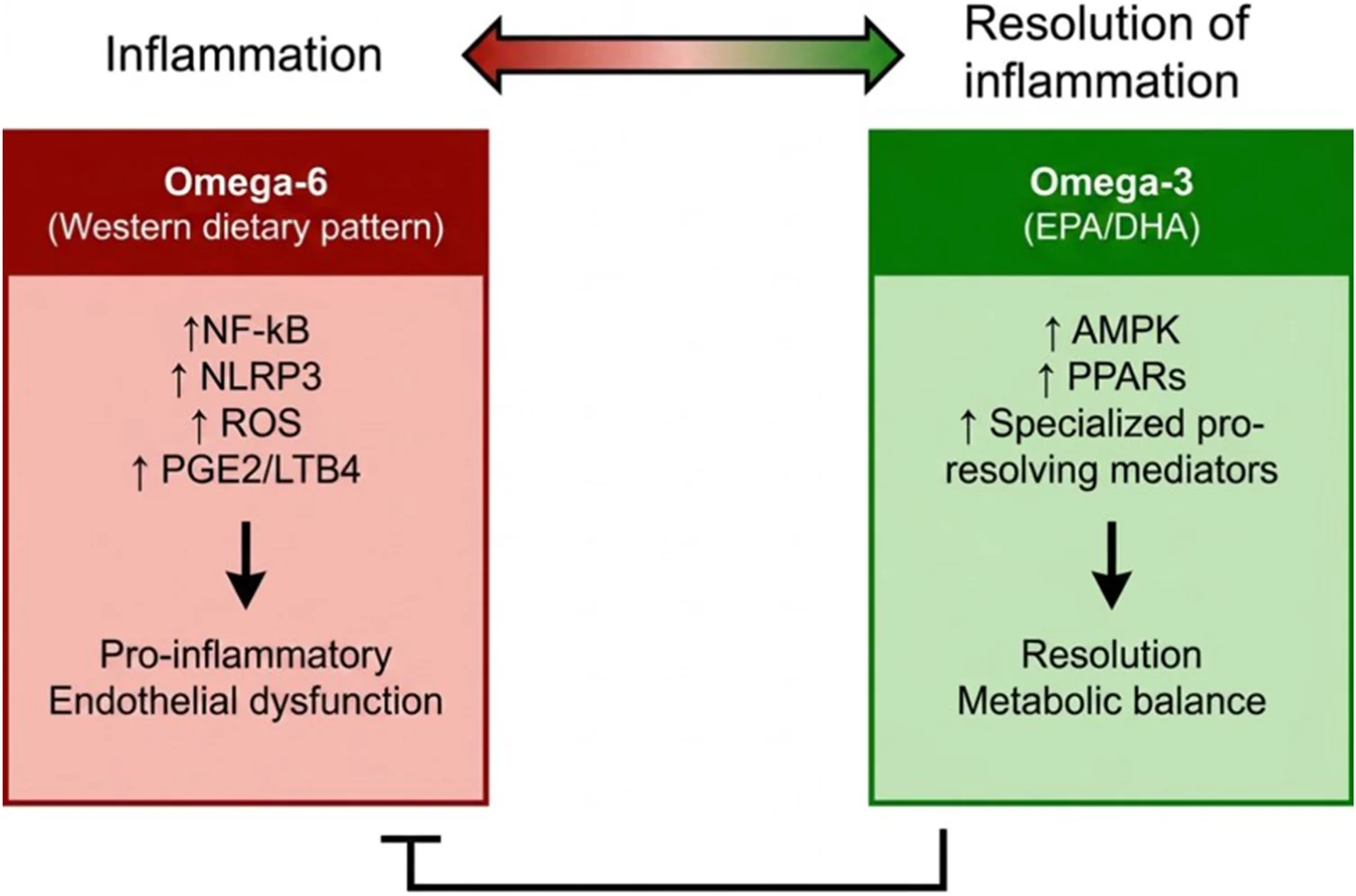
Balance between pro-inflammatory and pro-resolving effects of dietary fatty acids in immunometabolic regulation. Dietary fatty acids differentially regulate inflammatory and metabolic pathways. Omega-6 fatty acids, characteristic of Western dietary patterns, promote pro-inflammatory signaling through activation of NF-κB, increased production of reactive oxygen species (ROS), activation of the NLRP3 inflammasome, and synthesis of pro-inflammatory eicosanoids such as prostaglandin E2 (PGE2) and leukotriene B4 (LTB4). These pathways contribute to endothelial dysfunction and amplification of inflammation. In contrast, omega-3 fatty acids (EPA and DHA) activate anti-inflammatory and pro-resolving pathways, including AMP-activated protein kinase (AMPK) and peroxisome proliferator-activated receptors (PPARs), and serve as precursors of specialized pro-resolving mediators (SPMs). These mechanisms promote resolution of inflammation, restoration of metabolic homeostasis, and improved vascular function. The dynamic balance between omega-6 and omega-3 fatty acids influences immunometabolic outcomes during pregnancy.

Dietary intake is the primary determinant of long-chain omega-3 availability. Marine sources, especially fatty fish, provide preformed EPA and DHA, which are efficiently incorporated into cell membranes and influence downstream signaling processes. However, global intake of these nutrients remains suboptimal, with many populations failing to reach recommended levels of DHA, particularly during pregnancy (Calder, 2017; Cao et al., 2024; Calder, 2015).

### 3.2 Immunometabolic and placental mechanisms of UFAs in pregnancy

Pregnancy is characterized by dynamic metabolic adaptations that ensure adequate nutrient supply to the developing fetus. Early gestation is predominantly anabolic, favoring lipid accumulation, whereas late gestation shifts toward a catabolic state marked by increased lipolysis and circulating lipids. This physiological hyperlipidemia is accompanied by increased very low-density lipoprotein (VLDL) production and reduced clearance of triglyceride-rich lipoproteins (Geraghty et al., 2017; Ryckman et al., 2015).

The placenta plays a pivotal role in regulating maternal–fetal lipid transfer through the coordinated activity of lipases, fatty acid transporters, and intracellular binding proteins. This system is particularly important for DHA delivery, given its critical role in fetal neurodevelopment and membrane structure. In HRP, these regulatory mechanisms may become disrupted. Alterations in maternal lipid metabolism, together with oxidative stress and chronic low-grade inflammation, can impair placental lipid handling. This dysfunction promotes lipotoxicity, endothelial damage, and amplification of inflammatory responses, contributing to adverse outcomes such as fetal growth restriction, preeclampsia, and PTB (Gaccioli and Lager, 2016; Ryckman et al., 2015; Duttaroy and Basak, 2022; Cao et al., 2024; Aye et al., 2025).

At the molecular level, UFAs exert pleiotropic effects on cellular signaling and metabolic regulation. Changes in membrane lipid composition influence receptor function and downstream pathways, including nuclear factor kappa B (NF-κB), AMP-activated protein kinase (AMPK), and peroxisome proliferator-activated receptors (PPARs). AMPK acts as a cellular energy sensor that is activated under conditions of metabolic stress, promoting fatty acid oxidation and inhibiting energy-consuming processes, while also suppressing inflammatory signaling. In parallel, PPARs function as lipid-activated transcription factors that regulate genes involved in lipid metabolism, insulin sensitivity, and inflammation. Through coordinated regulation of these pathways, UFAs contribute to the integration of metabolic and immune responses (Calder, 2017; Liu et al., 2017; Hotamisligil, 2017; Djuricic and Calder, 2021; Policastro et al., 2023).

Omega-3 fatty acids modulate these processes by attenuating pro-inflammatory signaling, improving insulin sensitivity, and enhancing mitochondrial function. These effects are partly mediated through activation of AMPK and PPARs, as well as suppression of NF-κB–dependent transcriptional activity. Furthermore, omega-3 fatty acids influence inflammasome activity and oxidative stress, with experimental evidence demonstrating inhibition of NLRP3 activation, reduced generation of ROS, and improved endothelial function (Figure 3). These mechanisms are also highly relevant to the pathophysiology of spontaneous PTB, which is strongly associated with intrauterine inflammation and immune activation. Increased production of pro-inflammatory cytokines and eicosanoids, especially prostaglandins involved in uterine contractility and cervical ripening, represents a key trigger of preterm labor. By modulating inflammatory signaling and lipid mediator profiles, omega-3 fatty acids may counteract these processes (Calder, 2017; Yan et al., 2013; Serhan and Levy, 2018; Policastro et al., 2023; Thompson et al., 2023; Sousa et al., 2025; García-Morales et al., 2026).

**FIGURE 3:**
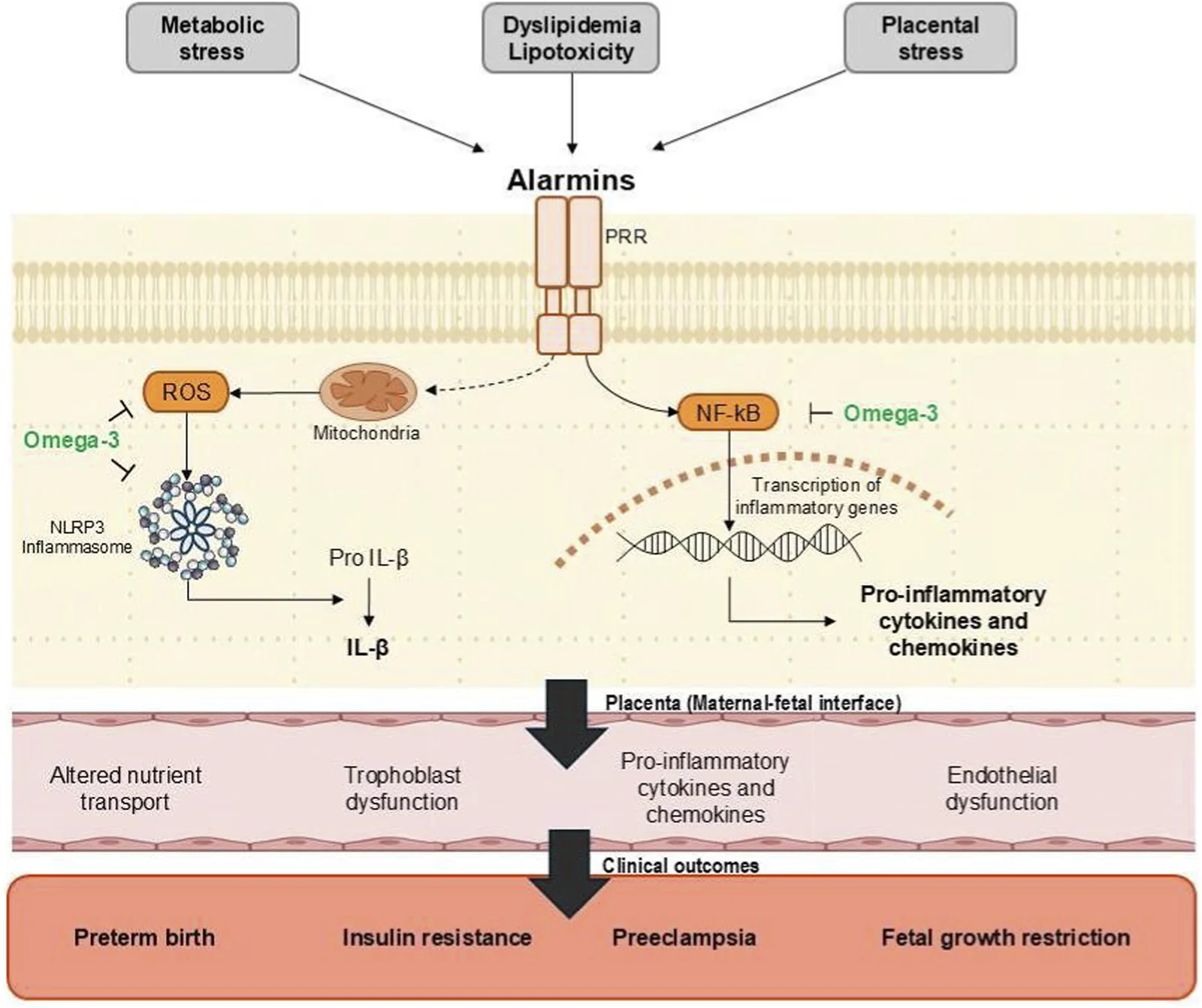
Immunometabolic mechanisms linking metabolic stress to placental dysfunction. Metabolic stressors, including dyslipidemia, lipotoxicity, and placental stress, trigger the release of endogenous danger signals (alarmins), which activate pattern recognition receptors (PRRs) on maternal and placental cells. This activation leads to downstream signaling through nuclear factor kappa B (NF-κB), promoting transcription of pro-inflammatory genes. Mitochondrial dysfunction increases the generation of reactive oxygen species (ROS), further amplifying inflammatory signaling and activating the NLRP3 inflammasome, resulting in the maturation and release of interleukin-1β (IL-1β). These processes enhance the production of pro-inflammatory cytokines and chemokines, contributing to trophoblast dysfunction, impaired nutrient transport, and endothelial dysfunction at the maternal–fetal interface. Omega-3 fatty acids exert modulatory effects by attenuating NF-κB activation, reducing ROS production, and inhibiting inflammasome activation, thereby promoting resolution pathways. Collectively, these mechanisms contribute to adverse clinical outcomes such as preterm birth, insulin resistance, preeclampsia, and fetal growth restriction.

A growing body of evidence from observational studies, randomized controlled trials (RCTs), and meta-analyses supports a role for omega-3 PUFAs, particularly DHA and EPA, in improving pregnancy outcomes (Table 1). Observational studies indicate that higher maternal intake or circulating levels of long-chain n-3 PUFAs are associated with reduced systemic inflammation and a lower risk of adverse outcomes, including PTB and impaired fetal growth (Olsen et al., 2018). Meta-analyses and systematic reviews consistently demonstrate reductions in PTB (<37 weeks) of approximately 10%–12% and in early PTB (<34 weeks) of up to 35%–40% (Middleton et al., 2018; Saccone and Berghella, 2015). Randomized trials further suggest that higher-dose DHA supplementation (approximately 600–1000 mg/day) may be particularly effective in women with low baseline DHA status, highlighting the importance of nutritional status as a determinant of treatment response (Makrides et al., 2010; Makrides et al., 2019; Carlson et al., 2021).

**TABLE 1:** Summary of human clinical evidence on Omega-3 fatty acid supplementation in high-risk pregnancies.

Study	Design	Intervention	Main outcomes	Key findings
Carlson et al., 2021	RCT	1000 mg vs. 200 mg DHA	Early PTB (<34 weeks): 1.7% vs. 2.4%; in women with low DHA status: 2.0% vs. 4.1%	Higher-dose DHA reduced early PTB, especially in women with low baseline DHA status
Makrides et al., 2019	RCT	800 mg DHA + 100 mg EPA	Early PTB: RR 1.13 (95% CI 0.79–1.63, p = 0.50)	No significant effect on early PTB or post-term interventions
Middleton et al., 2018	Meta-analysis	DHA/EPA (200–2700 mg/day)	PTB: RR 0.89 (95% CI 0.81–0.97) Early PTB: RR 0.58 (95% CI 0.44–0.77)	Evidence that omega-3 reduces the risk of preterm and early preterm birth
Olsen et al., 2018	Prospective cohort	Plasma n-3 levels	Early PTB: OR 10.27 (95% CI 6.80–15.5)	Women in the lowest EPA + DHA quintile had a 10-fold higher risk of early PTB.
Saccone and Berghella, 2015	Meta-analysis	Omega-3 supplementation	PTB: RR 0.90 (95% CI 0.72–1.11)	No significant reduction in preterm birth or improvement in neonatal outcomes
Zhou et al., 2012	RCT	Fish oil (800 mg DHA)	GDM: RR 0.97 (95% CI 0.74–1.27) PE: RR 1.12 (95% CI 0.84–1.51)	No significant reduction in GDM or preeclampsia

CRP, C-reactive protein; DHA, docosahexaenoic acid; EPA, eicosapentaenoic acid; GDM, gestational diabetes mellitus; PTB, preterm birth; RCT, randomized controlled trial.

In contrast, evidence for other outcomes remains less consistent. In preeclampsia, clinical trials have yielded conflicting results, and several well-conducted studies have not demonstrated significant reductions in disease incidence. Similarly, in GDM, Zhou et al. (2012) demonstrated that supplementation with 800 mg/day of DHA did not significantly reduce the incidence of GDM or preeclampsia, highlighting that potential improvements in metabolic and inflammatory pathways do not necessarily translate into clinically meaningful obstetric benefits. Beyond obstetric outcomes, omega-3 fatty acids have also been investigated in relation to maternal mental health and offspring neurodevelopment, although findings remain heterogeneous (Makrides et al., 2010; Reis et al., 2024). Overall, these data suggest that the effects of supplementation are context-dependent and influenced by baseline nutritional status, metabolic phenotype, and the timing and intensity of intervention.

## 4 Integrative immunometabolic model and translational perspectives

HRPs can be conceptualized as immunometabolic disorders in which metabolic stress, immune activation, and placental dysfunction interact within a self-reinforcing network that drives adverse maternal and fetal outcomes, including preeclampsia, GDM, fetal growth restriction, and PTB (Hotamisligil, 2017; Burton and Jauniaux, 2018; Rees et al., 2022; Aye et al., 2025).

In this context, UFAs, particularly omega-3 PUFAs, emerge as biologically relevant modulators of both inflammatory signaling and its resolution. By influencing membrane composition, intracellular signaling pathways, and the biosynthesis of specialized pro-resolving mediators, these lipids contribute to the restoration of tissue homeostasis and may attenuate placental and systemic inflammation (Calder, 2017; Innes and Calder, 2018; Serhan and Levy, 2018; Thompson et al., 2023; Sousa et al., 2025).

Although several studies support a beneficial role of omega-3 supplementation, the overall clinical evidence remains heterogeneous. While reductions in PTB are consistently observed, findings for other outcomes, such as preeclampsia and GDM, are inconsistent across randomized controlled trials. Differences in study design, baseline nutritional status, timing, dosage, and adherence likely contribute to these discrepancies (Middleton et al., 2018; Makrides et al., 2010; Makrides et al., 2019; Carlson et al., 2021; Zhou et al., 2012; Olsen et al., 2018; Saccone and Berghella, 2015).

Despite promising mechanistic insights and clinical evidence, several important limitations should be acknowledged. The literature is characterized by substantial heterogeneity in study design and participant characteristics, and many trials do not assess baseline omega-3 status. Adherence to supplementation protocols is often suboptimal and rarely confirmed by using objective biomarkers such as circulating fatty acid levels. Moreover, several rigorously conducted randomized trials have reported null findings, underscoring that omega-3 supplementation does not produce uniform effects across all populations and outcomes. Although omega-3 fatty acids are generally considered safe during pregnancy, the long-term effects and optimal upper limits of high-dose supplementation remain incompletely defined. Collectively, these limitations warrant cautious interpretation of the current evidence and reinforce the need for well-designed, biomarker-informed clinical trials.

Taken together, these findings support a transition from generalized supplementation toward biomarker-guided and individualized nutritional strategies. Pathophysiology-driven clinical trials incorporating standardized protocols, objective biomarkers of adherence and fatty acid status, and stratification according to baseline nutritional and metabolic profiles will be essential to identify the women most likely to benefit and to optimize the clinical application of omega-3 fatty acids in HRP (He et al., 2018; Santana et al., 2022; Savona-Ventura et al., 2024).

## 5 Conclusion

This review highlights unsaturated fatty acids, particularly omega-3 polyunsaturated fatty acids, as modulators of inflammatory and metabolic pathways involved in HRP. Clinical evidence is strongest for the prevention of PTB, especially among women with low baseline DHA status, whereas findings for preeclampsia and GDM remain inconsistent. Overall, current evidence supports a shift toward biomarker-guided and individualized supplementation strategies, while further mechanistically informed clinical trials are needed to optimize their application in high-risk pregnancies.
